# The Tea Plant Leaf Cuticle: From Plant Protection to Tea Quality

**DOI:** 10.3389/fpls.2021.751547

**Published:** 2021-10-01

**Authors:** Mingjie Chen

**Affiliations:** College of Life Sciences, Henan Provincial Key Laboratory of Tea Plant Biology, Xinyang Normal University, Xinyang, China

**Keywords:** *Camellia sinensis*, cuticle, wax, transpiration rate, transpiration barrier, correlation analysis, structure–function relation

## Abstract

*Camellia sinensis* (tea tree) is a perennial evergreen woody crop that has been planted in more than 50 countries worldwide; its leaves are harvested to make tea, which is one of the most popular nonalcoholic beverages. The cuticle is the major transpiration barrier to restrict nonstomatal water loss and it affects the drought tolerance of tea plants. The cuticle may also provide molecular cues for the interaction with herbivores and pathogens. The tea-making process almost always includes a postharvest withering treatment to reduce leaf water content, and many studies have demonstrated that withering treatment-induced metabolite transformation is essential to shape the quality of the tea made. Tea leaf cuticle is expected to affect its withering properties and the dynamics of postharvest metabolome remodeling. In addition, it has long been speculated that the cuticle may contribute to the aroma quality of tea. However, concrete experimental evidence is lacking to prove or refute this hypothesis. Even though its relevance to the abiotic and biotic stress tolerance and postharvest processing properties of tea tree, tea cuticle has long been neglected. Recently, there are several studies on the tea cuticle regarding its structure, wax composition, transpiration barrier organization, environmental stresses-induced wax modification, and structure–function relations. This review is devoted to tea cuticle, the recent research progresses were summarized and unresolved questions and future research directions were also discussed.

## Introduction

The cuticle is a hydrophobic coating on the aerial surface of all plants and serves as the interacting interface with the surrounding environment. Recently, a cuticle was also found from the surface of the root cap and lateral roots (Berhin et al., 2019). Plant cuticle protects the plant from dehydration or uncontrolled water absorption (Eigenbrode and Espelie, 1995), pathogen infection (Barthlott and Neinhuis, 1997), insect attack (Eigenbrode and Espelie, 1995), UV radiation (Reicosky and Hanover, 1978; Solovchenko and Merzlyak, 2003), and organ fusion (Weng et al., 2010; Ingram and Nawrath, 2017). The cuticle is a composite structure of polyester and waxes. The polymer matrix is constituted by cutin, cutan, and polysaccharides which form the cuticle backbone (Nawrath, 2006; Domínguez, et al., 2011a; Philippe et al., 2020a). Cutin consists of C16 and C18 hydroxy and epoxy–hydroxy fatty acids, and glycerol monomers (Kolattukudy, 2001; Nawrath, 2006; Pollard et al., 2008). Polysaccharides are found within the cuticular layer or throughout the cuticle (Guzmán et al., 2014a,b; Philippe et al., 2020b). The waxes are either overlaid on the outer surface of cutin matrix as epicuticular waxes (EWs) or embedded inside a cutin polyster network as intracuticular waxes (IWs; Jetter and Riederer, 2016). So far, the well-characterized wax components include very-long-chain fatty acids (VLCFAs) and their derivatives (alcohols, aldehydes, alkanes, ketones, and wax esters), flavonoids, and alicyclic compounds (triterpenoids, sterols, and tocopherols) (Post-Beittenmiller, 1996; Jenks and Ashworth, 2003; Kunst and Samuels, 2003; Samuels et al., 2008).

There are multiple excellent reviews on plant cuticle in existing literature (Eigenbrode and Espelie, 1995; Kerstiens, 1996a,b; Kunst and Samuels, 2003; Buchholz, 2006; Nawrath, 2006; Shepherd and Griffiths, 2006; Bird, 2008; Pollard et al., 2008; Samuels et al., 2008; Reina-Pinto and Yephremov, 2009; Bernard and Joubès, 2013; Lee and Suh, 2013; Hen-Avivi et al., 2014; Domínguez, et al., 2015; Fernandez et al., 2016; Fich et al., 2016; Ingram and Nawrath, 2017; Philippe et al., 2020a; Skrzydeł et al., 2021), and readers may consult them for further information. The scope of this review is to focus on recent research progresses in tea cuticle. The tea tree possesses several advantages as a model system for cuticle research. They are as follows: (1) it is well adapted to growth chamber or green house conditions; (2) it is a perennial evergreen shrub or tree, and new twigs can emerge year around which make research material readily available; (3) it can be clonally propagated to warrant material with same genetic background, which removes the effect of genetic variance on research results; (4) it has two different types of cuticles during leaf developments, which makes it unique to study cuticle development and evolution; (5) it is self-incompatible, with large genetic variations accrued during its evolutionary history through natural and artificial selection. Thousands of germplasms have been systemically collected, which provide a rich genetic resource to study cuticle structural–functional relationship as well as to discover new pathways for wax lipid biosynthesis. In the following pages, the recent progress in tea leaf cuticle research was first summarized, and then unresolved questions and future research directions were discussed.

## Tea Cuticle Surface Structure

**Figure 1:**
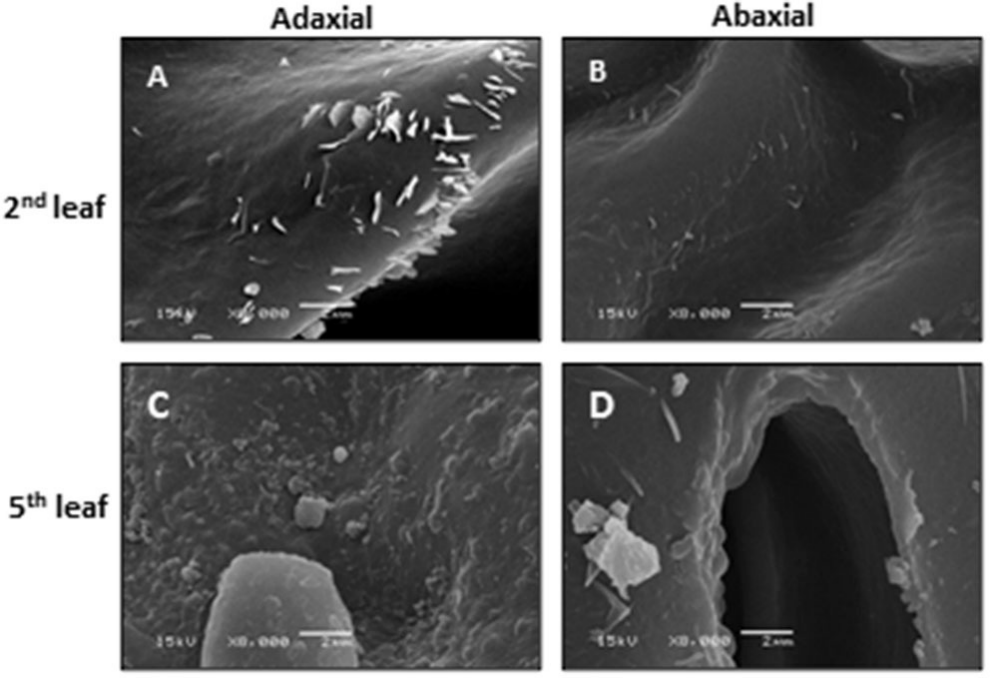
The scanning electron microscopy of the adaxial and abaxial surfaces of the second leaf and the fifth leaf. Bar = 2 μm. Modified from Chen et al. (2020). Scientific Reports 10: 6696.

Observed by scanning electron microscopy (SEM), the microcrystalline structure from the tea EWs shows three major shapes: rods, papilla, and plates. With tea leaf maturation, the sizes and shapes of wax crystals change accordingly. On tea buds, the wax crystals mainly show a papilla-like structure. The tender second leaf is dominated by rod-like crystals, whereas the mature fifth leaf is dominated by papilla- and plate-like wax crystals (Figure 1). Regardless of leaf position or maturity, more wax crystals are present on the adaxial surface compared with its abaxial surface. Koch and Ensikat (2008) demonstrated that crystal shapes are closely associated with wax compositions. Thus, the observed wax crystal changes during tea leaf maturation could reflect their wax chemical changes. The growth conditions also significantly affect the shape and density of the wax crystal. Water deprivation treatment significantly increases the density of the leaf wax crystal (Chen et al., 2020).

**Figure 2:**
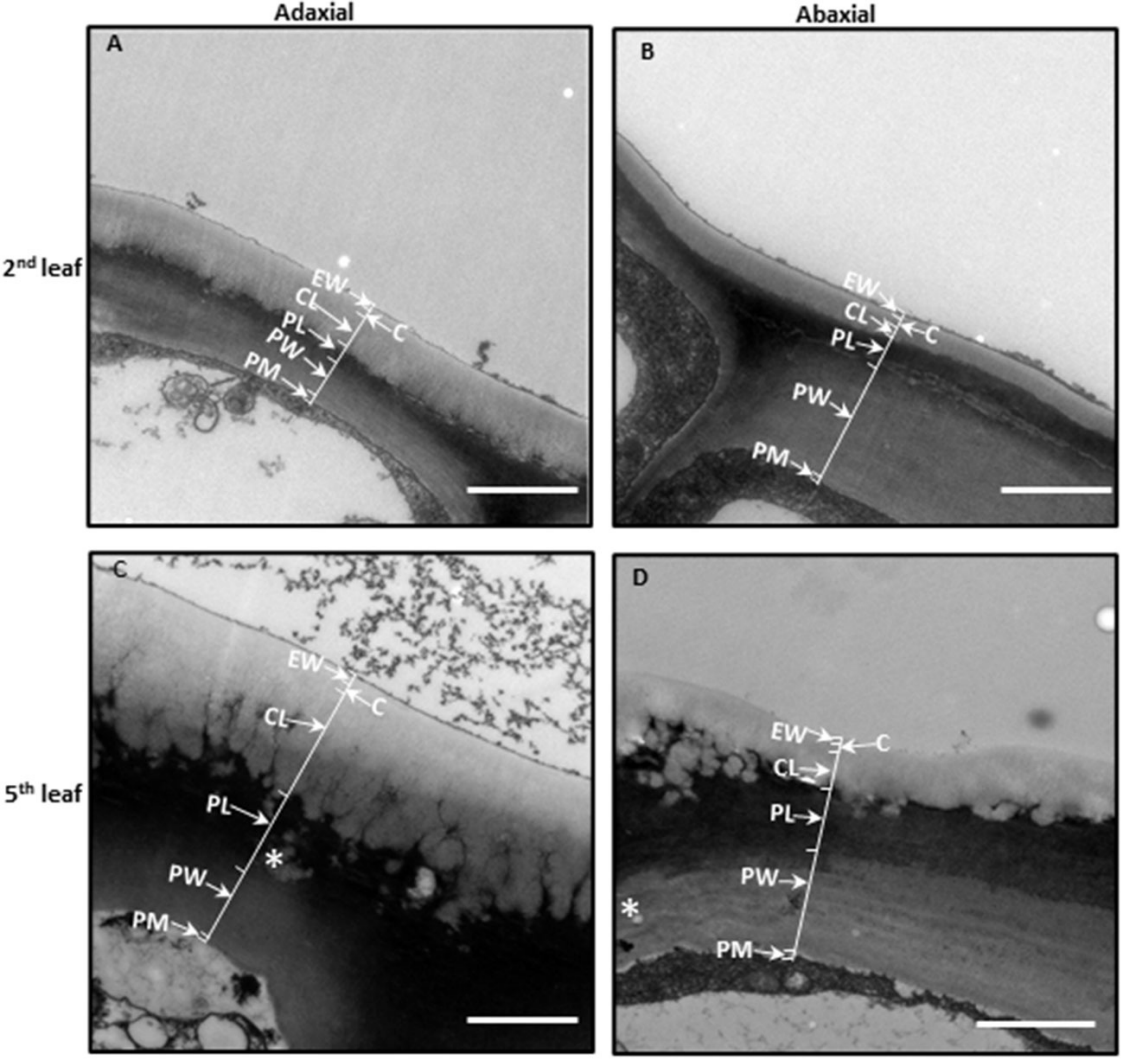
The leaf cuticle structure of *Camellia sinensis cv Fuyun 6*. **(A)** The adaxial side of the second leaf. **(B)** The abaxial side of the second leaf. **(C)** The adaxial side of the fifth leaf. **(D)** The abaxial side of the fifth leaf. Epicuticular waxes (EWs) cover the cuticle proper **(C)**, which is cutin embedded with intracuticular waxes (IWs). The cuticular layer (CL) seem also to contain intracuticular wax. Pectinaceous layer (PL), primary cell walls (PWs), and the plasma membrane (PM). Bar = 2 μm. The thickness of the cuticle was indicated by arrows. Cited from Zhu et al. (2018). Scientific Reports 8: 14944.

The cuticle thickness can be measured by SEM or transmission electric microscope (TEM). For the SEM method, the cuticle membrane needs to be isolated in advance. Jetter and Riederer (2016) applied this method and measured cuticle thickness from eight different plant species and found that the average cuticle thickness is in the range of 1–7 μm. Zhu et al. (2018) applied the TEM method to measure tea leaf cuticle thickness. Under the TEM image, the epidermal cell wall shows a layered configuration based on the staining patterns in an osmium tetroxide-uranyl acetate combination. Right under the cuticle, the cell wall is densely stained. In contrast, the epidermal cell wall facing the cytoplasm is only lightly stained. These differential staining patterns could reflect the compositional difference of the epidermal cell wall. Jeffree (2006) suggested that these densely stained layers are rich in pectin, and the constituent galacturonic acid moieties have free carboxyl groups which provide potential sites for ester linkages with cutin. Since the cuticle is just lightly stained by dyes like osmium tetroxide-uranyl acetate, there is a good contrast between the cuticle and cell wall, which makes the cuticle easily differentiated from cell wall in TEM imaging (Figure 2). Another advantage of the TEM method is that the cuticle thickness on the adaxial and the abaxial surface can be measured simultaneously. Zhang et al. (2021) applied the TEM method and measured the cuticle thickness of the fifth leaf from eight different tea germplasms, and the data were summarized in Table 1. The average thickness of the adaxial cuticle is in the range of 2.12–2.99 μm and the abaxial cuticle thickness is in the range of 1.24–1.46 μm. For individual tea germplasm, the adaxial cuticle generally is thicker than that of its abaxial counterpart.

**Table 1:** Cuticle thickness (μm) from the adaxial and the abaxial leaf surface of the eight tea germplasms.

	**Jinguanyin**	**0316B**	**Wuniuzao**	**0306A**	**0306H**	**Fuyun20**	**0202-10**	**Hongyafoshou**
Adaxial	2.99 ± 0.11^a^	2.42 ± 0.07^c^	2.12 ± 0.07^d^	2.77 ± 0.15^ab^	2.77 ± 0.04^ab^	2.56 ± 0.06^bc^	2.78 ± 0.08^ab^	2.65 ± 0.12^bc^
Abaxial	1.46 ± 0.04^a^	1.33 ± 0.05^ab^	1.37 ± 0.02^ab^	1.37 ± 0.06^ab^	1.24 ± 0.02^b^	1.45 ± 0.05^a^	1.36 ± 0.05^ab^	1.31 ± 0.02^b^

*Different lower-case letters represent statistically significant (p < 0.05). Data are expressed as mean ± SE (n = 6)*.

The TEM method also enables measuring the thickness of the EW layer and the IW layer. Zhu et al. (2018) follow up on the changes of cuticle thickness during tea leaf maturation. In the rapidly growing tea twig, the closer to the apical bud, the more tender the leaf is. Thus, the authors use the leaf position as a proxy for leaf maturity. The cellular characteristics also demonstrated that the second leaf from the apical bud is immature, and the fifth leaf becomes fully mature. In *Camellia sinensis cv Fuyun 6*, the adaxial cuticle thickness of the second leaf was 1.15 μm, and the average thickness of the EW layer and the IW layer was 0.19 and 0.97 μm, respectively; the average abaxial cuticle thickness was 0.47 μm, and the EW layer and the IW layer account for 0.13 and 0.34 μm, respectively. Although the total cuticle thickness of the fifth is doubled, the EW layer is only slightly increased, suggesting that the majority of the increase in cuticle thickness is attributed to the IW layer (Zhu et al., 2018).

The morphology of each cuticular layer also shows differential changes. The epicuticular layer facing the outer surface maintains a straight outline during leaf maturation; however, the IW layer facing the cell wall shows dramatic morphological changes with leaf maturation. The initial straight lines disappeared and were substituted by many small ridges, between ridges channels also are visible. During leaf maturation, the adaxial cuticle showed more pronounced structural changes compared with the abaxial cuticle (Figure 2).

## Tea Leaf Cuticular Wax Composition and Distribution Patterns

Chemical analysis demonstrates that tea leaf cuticular waxes are constituted by 14 chemical classes, including acids, 1-alkanols, aldehydes, alkanes, 1-alkanol esters, glycol esters, benzyl esters, phenethyl esters, phthalate esters, glycols, tocopherols, triterpenoids, sterols, and caffeine (Zhu et al., 2018; Zhang et al., 2020). It is well documented that the amounts and composition of cuticular waxes vary greatly among plant species, organs, tissues, or even developmental stages (Barthlott and Neinhuis, 1997; Jetter et al., 2007; Kosma et al., 2010; Buschhaus and Jetter, 2011; Bernard and Joubès, 2013). To offer a more detailed overview about wax distribution patterns, Zhang et al. (2020) isolated the EWs and the IWs from the adaxial and the abaxial surface, and then quantified the wax coverage and compositions. The wax coverages from the adaxial and the abaxial surfaces do not correlate with their respective cuticle thickness. Although the adaxial cuticle is thicker than that of the abaxial cuticle, its wax coverage is similar to the abaxial surface. On the adaxial surface, the waxes are almost equally distributed between EWs and IWs; however, on the abaxial surface, the IWs coverage is about 1.45–3.33 times higher than that of the EWs. For individual germplasms, the coverage of the adaxial EWs generally was higher than that of the abaxial EWs. Among the four cuticular compartments, the coverage of the abaxial IWs ranked as the highest among all the tea germplasms studied. By combining the cuticle thickness and the wax coverage data, the wax density from the adaxial and the abaxial surface is obtained. The EW density from the adaxial and the abaxial surfaces are 17.4 ± 1.0 and 19.7 ± 1.4 mg cm^−3^, respectively; the IW density from the adaxial and the abaxial surface are 3.9 ± 0.1 and 7.8 ± 0.2 mg cm^−3^, respectively. Overall, the wax density from the adaxial surface is lower than that of the abaxial surface.

Compared to the abaxial EWs, the adaxial EWs showed higher coverage of aldehydes, 1-alkanols, alkanes, and β-tocopherol. Compared to the abaxial IWs, the adaxial IWs showed higher coverage of 1-alkanols, alkanes, and β-tocopherol, but lower coverage of triterpenoids, steroids, and caffeine (Chen et al., 2021). Due to these unsymmetrical distributions of individual wax components on both leaf surfaces, the adaxial coverages of 1-alkanols, alkanes, and β-tocopherol are higher than that of the abaxial surface; in contrast, the adaxial coverage of triterpenoids, steroids, and caffeine are lower than that of the abaxial surface. The unsymmetrical deposition of cuticular wax into the adaxial and the abaxial surfaces may have ecophysiological implications for these two leaf surfaces. However, the underlying mechanisms to shape such wax distribution patterns remain unclear.

## Cuticular Wax Transport

All the wax components are synthesized in the endoplasmic reticulum (ER) of epidermal cells (Li-Beisson et al., 2010). After synthesis in the ER, waxes need to be transported to the plasma membrane (PM) for export. It is widely accepted that there are three possible routes for wax transportation from ER to PM: (1) Golgi and trans-Golgi network-mediated vesicle trafficking (Kunst and Samuels, 2003); (2) transport by cytosolic carrier proteins such as acyl-CoA binding proteins (Leung et al., 2006; Xiao and Chye, 2009; Xue et al., 2014); and (3) direct transfer through ER–PM membrane contact sites (Levine, 2004; Bernard and Joubès, 2013).

For the translocation of cuticular waxes from the PM to the apoplast, ATP-binding cassette transporter family proteins including ABCG1, 2, 6, 11, 12, 13, 20, and 32, play important roles (Vishwanath et al., 2015; Fich et al., 2016; Shanmugarajah et al., 2019). ABCG11 forms heterodimer with ABCG12 and is required for ABCG12 trafficking to the PM (Pighin et al., 2004; Bird et al., 2007; Luo et al., 2007; Panikashvili et al., 2007; Ukitsu et al., 2007; McFarlane et al., 2010). Since the ABCG11 and ABCG12 double mutants still retain ~50% surface wax (Bird et al., 2007), the other ABCG transporters are expected to be involved in wax secretion.

Lipid transfer proteins have been suggested to be involved in the transport of hydrophobic cuticular lipids from the PM through the hydrophilic cell wall and to the cuticle (Somerville et al., 2000). One member of this family, glycosylphosphatidylinositol-anchored lipid transfer protein (LTPG), is localized to the exterior face of the PM and may be involved in wax transport (Sterk et al., 1991; Thomas et al., 1993; Kunst and Samuels, 2003; DeBono et al., 2009; Lee et al., 2009). Since LTPG has a glycosylphosphatidylinositol (GPI) anchor to move across the cell wall with wax cargo, the GPI anchor needs to be cleaved first; alternatively, LTPG could have a different function in wax export.

**Figure 3:**
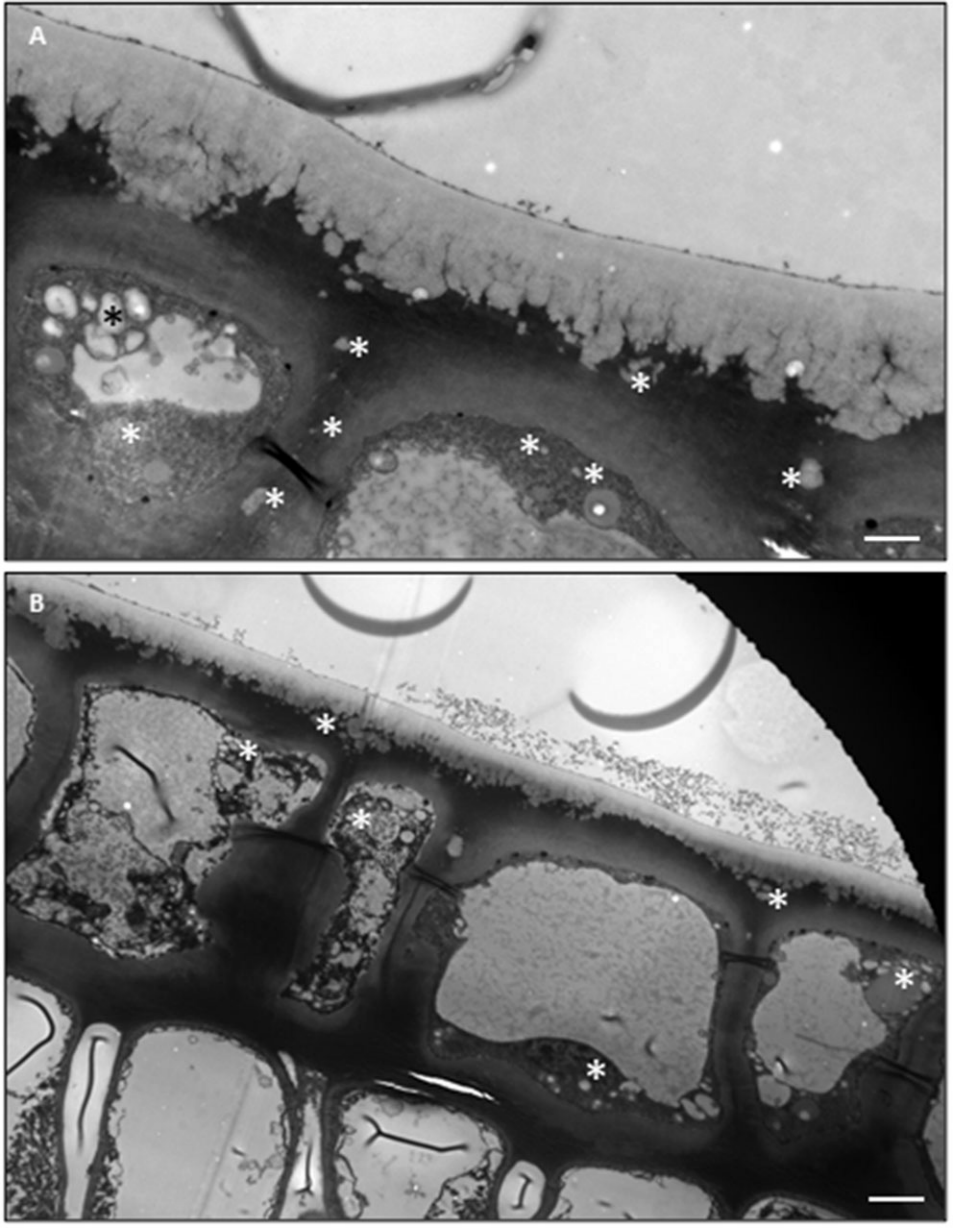
TEM imagines of the adaxial and the abaxial epidermal cells and the underlying palisiad cells. **(A)** The adaxial surface. **(B)** The abaxial surface. Bar = 2 μm.

Under the observation of TEM, the epidermal cells are filled with 1–2 μm whitish drops, which are not observed in the underlying palisade cells (Figures 3A,B). These drops are suggested to be lipid inclusion bodies of oleophilic droplets (Hoffmann-Benning et al., 1994). Similar size of inclusion bodies is also observed within the periclinal pectin layer of the cell wall as well as the anticlinal pectin layer which is formed by two neighboring epidermal cells (Figures 3A,B). These observations raise the possibility that cuticular wax could be transported from ER to PM in the form of inclusion bodies. Once reaching PM these inclusion bodies could be exported into the apoplast by exocytic vesicles and then diffuse through the pectin layer of the cell wall, until they reach and superimpose into the existing cuticle facing the protoplast surface. In deep-water rice and sorghum, osmiophilic vesicles near to or fusing with the PM have been reported before (Hoffmann-Benning et al., 1994; Jenks et al., 1994; Jeffree, 2006; Pollard et al., 2008), suggesting that such wax transportation mechanisms could exist in multiple plants. However, the structure and chemical composition of these inclusion bodies in tea and other plants remain unresolved.

## Developmental and Environmental Modification of Tea Cuticle and Cuticular Wax

Based on the presence and percentage of triterpenoids, plant cuticle can be divided into two types: the cuticle mainly is composed of VLCFAs and their derivatives, and the cuticle contains a high percentage of triterpenoids besides VLCFAs (Jetter and Riederer, 2016). Zhu et al. (2018) and Chen et al. (2020) found that the cuticular waxes from the juvenile (tender) leaf are mainly constituted by VLCFAs and their derivatives; in contrast, the mature leaf cuticle contains high levels of triterpenoids in addition to VLCFAs. Similar observation was recently reported in *Sorghum bicolor* (Busta et al., 2021). By now, it still remains unclear when the leaf cuticle makes such a transition and what are the underlying mechanisms. The analysis of cuticular waxes from each leaf position of a growing twig would clarify this issue. It has been reported that in other plant species, oxidosqualene cyclase (OSC) genes control the accumulation of cuticular triterpenoids (Thimmappa et al., 2014). The cuticular wax chemistry transition during tea leaf maturation could also be associated with *CsOSCs* expression. It will be interesting to investigate how many isoforms of *CsOSCs* are present in the tea genome, and how they are activated in epidermal cells during tea leaf maturation and contribute to triterpenoid accumulation in the leaf cuticle.

Cuticle structure and wax composition can also be affected by environmental factors such as drought (Chen et al., 2020; Zhang et al., 2021). Even though the tender leaves and the mature leaves have profound differences in cuticular wax chemistry, drought stress induces both types of leaves with some common compositional changes in cuticular waxes. For example, the wax coverage, cuticle thickness, and osmiophilicity of the tender leaf and mature leaf are increased, and some new wax species are synthesized and existing wax profiles are commonly modified (Chen et al., 2020).

**Figure 4:**
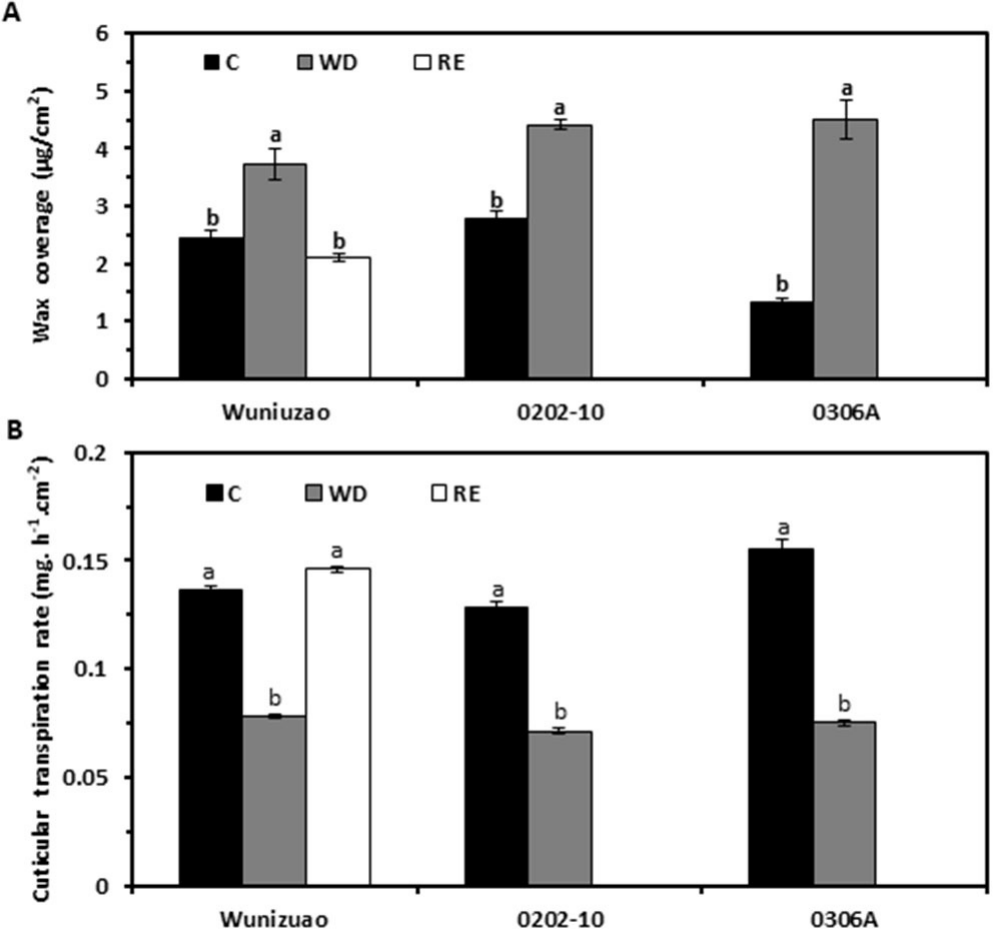
The water deprivation and rehydration treatments alter wax coverage and cuticular transpiration rate dynamically in three tea germplasms. **(A)** Wax coverage changes under different growth conditions. **(B)** Cuticular transpiration rate changes under different growth conditions. C: control; WD: water deprivation; RE: rehydration. Statistically significant changes within the same germplasm under different treatments were indicated by different letters. Modified from Zhang et al. (2021). Frontiers in Plant Science 11: 600069.

**Figure 5:**
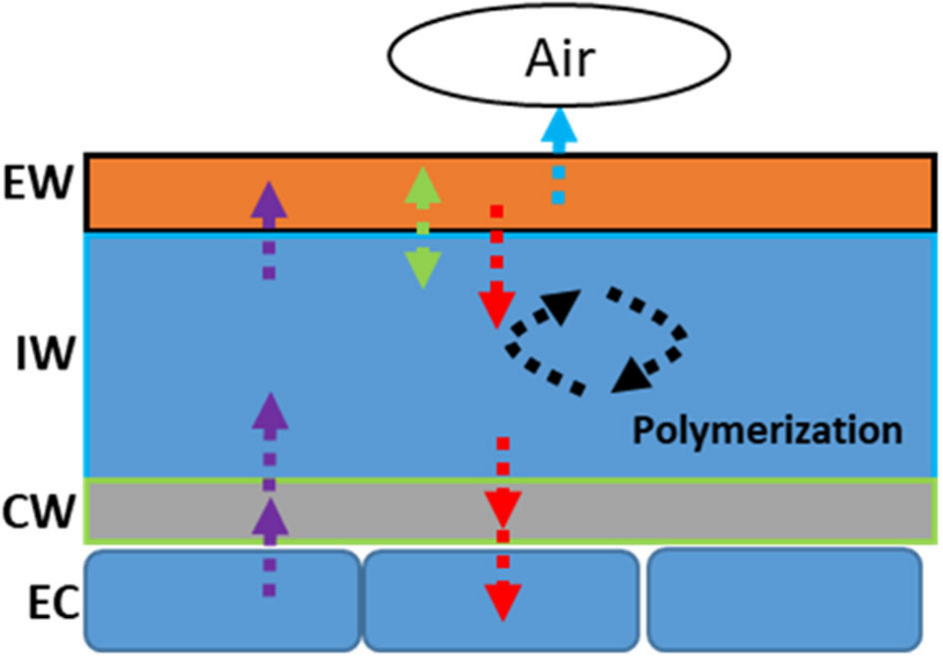
Schematic representation of the modification modes of the cuticular wax in response to drought stress. EW: epicuticular waxes; IW: intracuticular waxes; CW: cell wall; EC: epidermal cell.

The life span of tea leaf is around 1 year; it is expected that tea leaves will experience annual rain season and dry season. Since the cuticle is generally regarded as a nonliving tissue, the question is whether the cuticle modification is reversible once the stress conditions (such as drought) have disappeared. Zhang et al. (2021) reported that wax coverage and cuticular transpiration barriers indeed are reinforced by drought, but can be reversed following rehydration treatment (Figures 4A,B). There are multiple potential nonexclusive mechanisms working together to regulate cuticular wax deposition, including *in vivo* wax synthesis or transport in epidermal cells, dynamic phase separation between the EWs and the IWs, *in vitro* polymerization (Spencer et al., 1988), and retro transportation into epidermal cell wall or cytoplasm for further transformation (Figure 5; Zhang et al., 2021).

## Tea Leaf Cuticular Transpiration Rate Measurement

The principal function of the cuticle is to serve as a water transpiration barrier. The cuticular transpiration measurement provides essential information regarding its barrier properties. Several different methods have been developed in the past, including microbalance (Schönherr and Lendzian, 1981), ^14^C-epoxiconazole tracer permeability assay (Ballmann et al., 2011), and specially designed transpiration chamber (Becker et al., 1986). Different detection technologies were also developed, including the electrolysis cell (Keidel, 1959), moisture sensor, and ^3^H-labeled water in combination with a scintillation counter (Schreiber et al., 2001). Cuticular membrane isolation method, based on enzyme digestion, was also developed (Schönherr and Riederer, 1986). These technical advances facilitate the cuticular transpiration measurement (Becker et al., 1986). A common limitation of these methods is that only isolated cuticular membrane can be applied; in addition, the isolated cuticular membrane must be stomata-free. To overcome these limitations, Zhang et al. (2020) developed a new method that can measure the cuticular transpiration rates from the excised intact leaf; in addition, the transpiration from the adaxial and the abaxial surface can be obtained simultaneously. To make this measurement method robust and reproducible, several pretreatments are required: (1) Water equilibration: To minimize the influence of leaf water content variations on transpiration rate measurement, the excised twigs are equilibrated in water overnight to make leaf water contents even. (2) Close stomata: The stomata have a large influence on the cuticular transpiration rate measurement and it is desirable to make the stomata closed fully. For this purpose, three different treatments are applied: (1) the water equilibration be performed under the dark condition to promote stomata closure; (2) before leaf excision for transpiration measurement the ABA is applied to promote stomata closure. The data demonstrated that for the fully equilibrated leaf these combined treatments still cannot make stomata close fully. However, dehydration treatment for 4–5 h following leaf excision can effectively make stomata close, thus a constant minimal transpiration rate can be reached (Burghardt and Riederer, 2003; Zhang et al., 2020). Based on this phenomenon, the residual stomata transpiration can be estimated by using the total leaf transpiration difference between 0 and 5 h after leaf excision. The minimal transpiration (T) can be obtained from the leaf drying curve after the stomata became fully closed. (3) Vaseline application. Vaseline can be applied onto leaf abaxial surface to block stomata transpiration.

The total leaf transpiration rate (T) of control can be expressed as: (I) T = T_Ad_ + T_Ab_, where T_Ad_ and T_Ab_ represent the adaxial and the abaxial leaf transpiration rates, respectively. The adaxial transpiration rate can be regarded as equivalent to the adaxial cuticular transpiration rate. Due to the presence of stomata, the abaxial transpiration rate (T_Ab_) is the sum of the abaxial cuticular transpiration rate (T_Ab_c_) and the residual stomatal transpiration rate (T_Ab_s_). A mathematical relationship can be expressed as: (II) T_Ab_ = T_Ab_c_ +T_Ab_s_.

Using vaseline to seal the leaf surface can effectively reduce water transpiration. When both leaf surfaces are sealed with vaseline, the observed leaf transpiration rate (T_Ad/Vas::Ab/Vas_) is equivalent to total leaf transpiration rate (T) multiplied by a vaseline diffusion coefficient factor k: (III) T_Ad/Vas::Ab/Vas_ = k × T. Since T_Ad/Vas::Ab/Vas_ and T can be experimentally measured from the control and the Vaseline-treatment group, the k value can be calculated. Theoretically, k is a parameter related to the applied vaseline film and affected by the film thickness and evenness; Thus it should be a constant once a stable vaseline film is established. However, after leaf excision, the observed total transpiration rate T of the control gradually decreased whereas the observed total transpiration rate from vaseline-sealed group kept fairly stable, which resulted in the k value increasing gradually. The decrease in the total transpiration rate of the control plants likely results from the gradual stomata closure induced by leaf dehydration following excision. A stable k can be obtained after stomata closure fully, which takes about 4–5 h following tea leaf excision.

When the adaxial surface is sealed with vaseline (Ad/Vas), the observed total transpiration rate (T_Ad/Vas_) is given by the formula: (IV) T_Ad/Vas_ = k × T_Ad_ + T_Ab._

Similarly, when the abaxial surface is sealed with vaseline (Ab/Vas), the observed total transpiration rate (T_Ab/Vas_) is given by the formula: (V) T_Ab/Vas_ = T_Ad_ + k × T_Ab._

**Figure 6:**
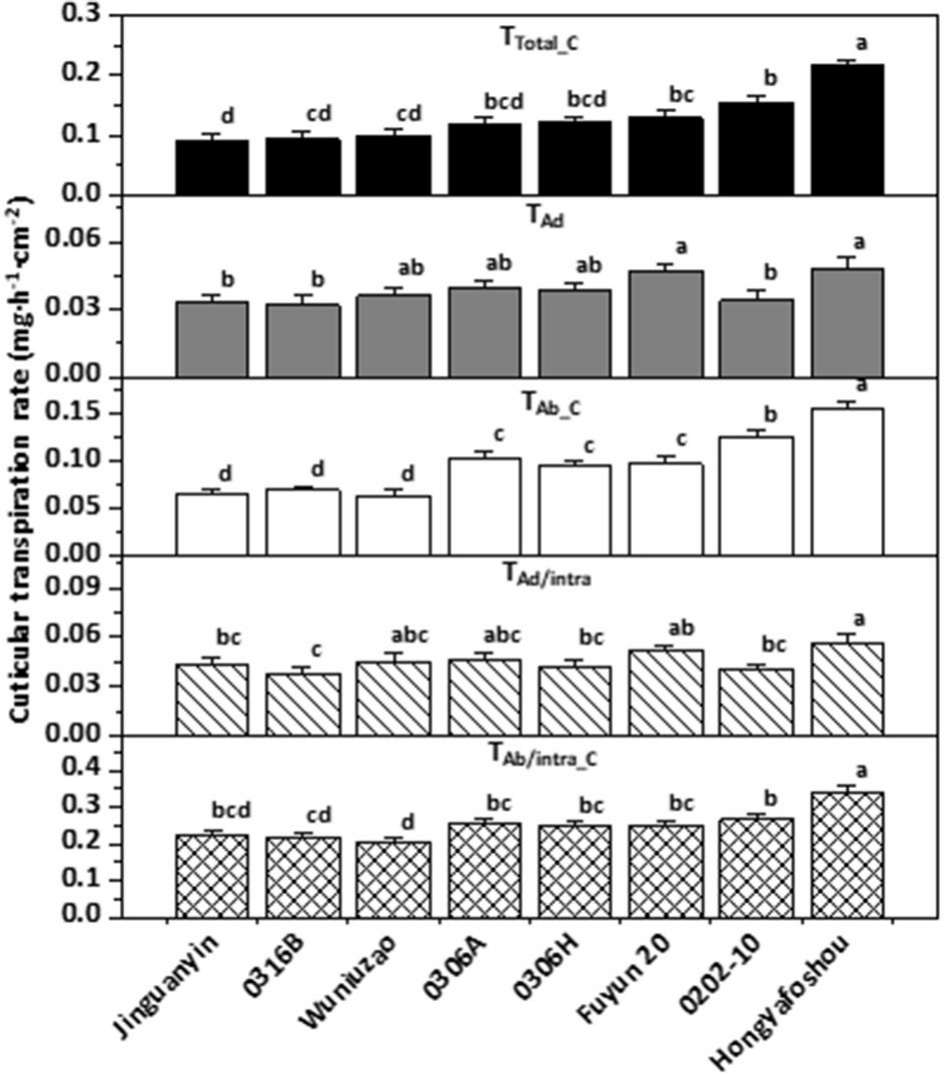
The cuticular transpiration rates of the eight tea germplasms. Different lower-case letters represent statistically significant (P *P* < 0.05). TTotal_C: the total cuticular transpiration rate; TAd: the adaxial cuticular transpiration rate; TAb_C: the abaxial cuticular transpiration rate; TAd/intra: the adaxial intracuticular transpiration rate; TAb/intra_C: the abaxial intracuticular transpiration rate. Modified from Chen et al. (2021). Frontiers in Plant Science 12: 655799.

Based on the observed value for T, T_Ad/Vas::Ab/Vas_, T_Ad/Vas_, T_Ab/Vas_, the mathematical formula I–V, the cuticular transpiration rates from the adaxial, and the abaxial leaf surface can be calculated. Zhang et al. (2020) found that the abaxial cuticular transpiration rate is about 1-fold higher than that of the adaxial cuticular transpiration rate in *C. sinensis cv Fuyun 6*. To test if this is a common or just a germplasm-specific phenomenon, Chen et al. (2021) measured the cuticular transpiration rates from other eight tea germplasms and found that the abaxial cuticular transpiration rates showed much larger variations among these tea germplasms and was about 1.8–3.3 folds higher than that of the adaxial surface; in contrast, the adaxial cuticular transpiration rates showed much smaller variations across diverse germplasms (Figure 6). These results demonstrated the unsymmetrical distribution of the cuticular transpiration barrier between the adaxial and the abaxial surfaces.

## Cuticular Transpiration Barrier Organizations on the Adaxial and the Abaxial Leaf Surfaces

**Figure 7:**
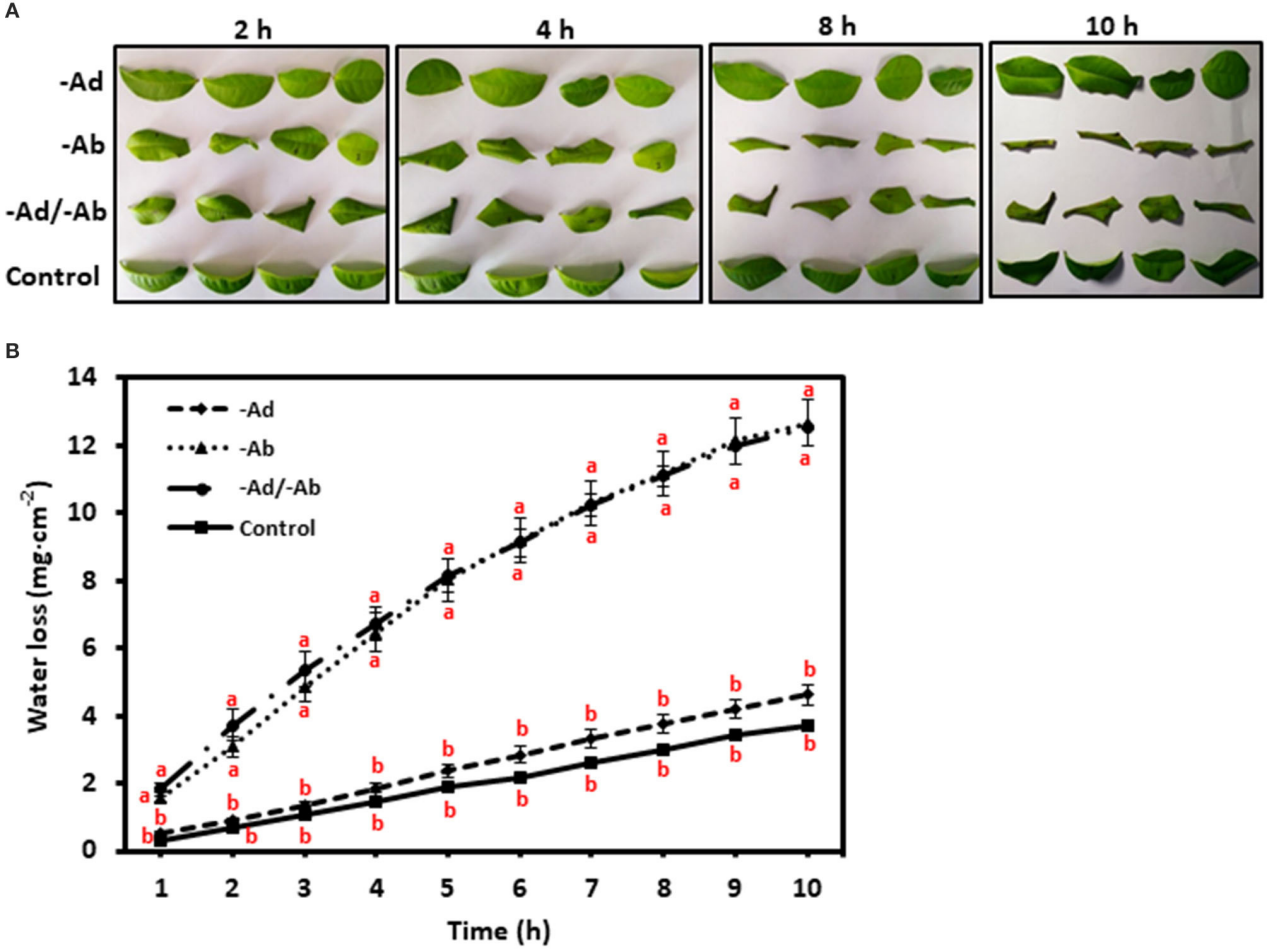
**(A,B)** Adaxial and abaxial epicuticular waxes (EWs) had different contributions to the leaf transpiration barrier. –Ad, adaxial EW waxes were removed by gum Arabic; –Ab, abaxial EW waxes were removed by gum Arabic; –Ad/–Ab, EW waxes from both surfaces were removed by gum arabic. Data are expressed as means ± standard error (*n* = 5). Statistical analysis was performed among different treatments at same time point, and different letters at the same time point indicate statistically significant (p<0.05). Cited from Zhang et al. (2020). Frontiers in Plant Science 11: 420.

It has been demonstrated that cuticular waxes rather than cutin matrix make significant contributions to the cuticular transpiration barrier (Schönherr, 1976; Vogg et al., 2004; Jetter and Riederer, 2016; Sadler et al., 2016). Since cuticular waxes can be divided into EWs and IWs (Jetter and Riederer, 2016), the question is which one, the EWs, the IWs, or both contribute to the cuticular transpiration barrier? To answer this question, a simple way is to selectively remove EWs and then measure the leaf transpiration changes. If EWs removal significantly increases the leaf transpiration rate, then this would suggest that these EWs play an important role in shaping the surface transpiration barrier. If EWs removal does not affect the transpiration rate, this would suggest that these EWs do not contribute much to the transpiration barrier. Previous work has shown that gum Arabic can selectively and efficiently strip off EWs without affecting IWs (Jetter and Schäffer, 2001; Jetter and Riederer, 2016; Zeisler and Schreiber, 2016). Zeisler and Schreiber (2016) and Zeisler et al. (2018) studied 10 different plant species including the tea tree, and the authors concluded that the adaxial EWs are not the main cuticular transpiration barrier, instead, the adaxial IWs constitute the main cuticular transpiration barrier. This conclusion is independently confirmed by Zhang et al. (2020) who applied a different measurement method. However, the transpiration barrier organization for the abaxial cuticle remains largely unresolved due to the technical hurdles. The established new method by Zhang et al. (2020) paved the way to measure the abaxial cuticular transpiration rate. The data demonstrated that the abaxial EWs constitute a major cuticular transpiration barrier, whereas the abaxial IWs do not (Figure 7). Correlation analysis from the adaxial and the abaxial surface suggest that the differential distribution of VLCFAs, 1-alkanol esters, and glycols may contribute to the unsymmetrical cuticular transpiration barrier on both leaf surfaces (Zhang et al., 2020). By now, it still remains unclear what the ecobiological implications for the differential organization of cuticular transpiration barrier on the two surfaces of the leaves are. We speculate that the adaxial surface is optimized to restrict water loss, whereas the abaxial surface could be evolved to cope with other environmental stresses, which compromise its potency as an efficient water transpiration barrier. Since the adaxial and the abaxial cuticles are independently synthesized by the adaxial and the abaxial epidermal cells, respectively, and the adaxial epidermal cells and the abaxial epidermal cells have different niches (such as light irradiance), differential regulations of wax synthesis or transport from the adaxial and the abaxial epidermal cells may occur, and this would eventually affect wax composition on the EWs and the IWs of the adaxial and the abaxial cuticle.

## Cuticular Waxes that Affect Cuticular Transpiration Rate or Resistance Under Normal and Stress Conditions

Cuticular waxes from individual cuticular compartments can be isolated and their coverage and composition is measured by GC-MS and GC-FID. Cuticular transpiration rates or resistance can also be determined from individual cuticular compartments. The correlation analysis between wax chemistry and cuticular transpiration offers a powerful tool to identify contributing wax components to cuticular transpiration barrier properties. This brings up two questions: (1) Are the contributing waxes for the transpiration barrier affected by plant growth conditions? (2) Does the wax subcompartment localization affect its contribution to the transpiration barrier property? To address these questions, Zhang et al. (2020) grew three tea germplasms in a greenhouse. Water was withheld for a certain period to induce drought stress, and then water was resumed for the recovery of plants. The EWs and the IWs from the adaxial and the abaxial surfaces of the fifth leaf were isolated and quantified, whereas the cuticular transpiration rates were measured from leaf drying curves. The data showed that the cuticular transpiration barriers were enhanced by drought stress, and the initial weak cuticular transpiration barriers were preferentially reinforced, an effect known as “make up for shortcomings,” and rehydration treatment lessened the cuticular transpiration barrier. Thus, the cuticular transpiration barrier can be reversibly modified depending on the growth conditions of plants. Correlation analysis demonstrated that the modification of the cuticular transpiration barrier does not require an overhaul of all wax components, instead targeted deposition of some specific wax compounds into individual cuticular compartments is sufficient to alter the cuticular transpiration barrier properties (Table 2).

**Table 2:** Correlations (Pearson's *R*^2^ values) between cuticular transpiration rate and a different compound class of cuticular waxes.

	**Adaxial surface**		**Abaxial surface**	
	**EW**	**IW**	**EW**	**IW**
Acides	−0.51	−0.25	−0.07	−0.54
Aldehydes	−0.68*	−0.59*	−0.14	+0.03
1-Alkanols	−0.39	−0.32	−0.07	−0.93*
Alkanes	−0.13	+0.06	−0.01	+0.01
1-Alkanol esters	−0.59*	+0.09	−0.08	+0.08
Glycol esters	−0.52	−0.00	−0.33	−0.12
Phthalate esters	−0.39	+0.15	−0.39	+0.26
Glycols	−0.00	+0.42	−0.01	−0.03
β-Tocopherol	−0.37	−0.04	−0.07	−0.01
Triterpenoids	−0.53	−0.71*	+0.01	−0.88*
Steroids	−0.00	−0.07	+0.07	−0.74*
Caffeine	−0.50	+0.00	−0.36	+0.00
Subtotal coverage	−0.51	−0.78*	−0.11	−0.97*

*Plus (+) and minus (–) represent positive and negative correlation with minimum transpiration rate. Values labeled with asterisk are significant at P < 0.05. EW, epicuticular wax; IW, intracuticular wax*.

*Cited from Zhang et al. (2021)*.

In literature, it has been controversial regarding alicyclic compounds (triterpenoids and steroids) for their contributions to the cuticular transpiration rate. The triterpenoids were reported to be positively correlated with cuticular transpiration rates. Thus they were not regarded as contributors to the cuticular transpiration barrier (Vogg et al., 2004; Buschhaus and Jetter, 2012; Jetter and Riederer, 2016), whereas in other reports they were identified as a contributor to the cuticular transpiration barrier (Schuster et al., 2016; Zeisler and Schreiber, 2016; Romero and Rose, 2019). Zhang et al. (2021) found that under drought stress the triterpenoids and steroids from IWs were negatively and significantly correlated with cuticular transcription rate, suggesting that they contribute to the cuticular transpiration barrier under drought conditions. Under the described experimental conditions, tea plants were also subjected to high temperature and high light radiation stresses besides dehydration stress (Zhang et al., 2021). To cope with these multiple stresses, alicyclic compounds could serve as cuticle nanofiller to increase cuticle stiffness and breaking stress and decrease its maximum strain. Consistent with this notion, under normal growth conditions, triterpenoids and steroids were not found to be correlated with cuticular transpiration rate (Chen et al., 2021). Recently, Busta et al. (2021) compared the leaf wax divergence between sorghum and maize, and they identified an OSC gene (Sobic.008G142400.1) which is involved in generating the majority of sorghum leaf surface triterpenoids. These triterpenoids are major components of the IW layer on both sides of the leaf. They could act as strengthening nanofillers (Tsubaki et al., 2013) and contribute to the performance of the leaf cuticle at elevated temperatures (Schuster et al., 2016). These findings collectively support the notion that cuticle triterpenoids play important roles under stress conditions rather than normal growth conditions (Chen et al., 2021). It remains an open question whether triterpenoids play other critical functions (such as defense) under normal growth conditions. Considering that such a large quantity of triterpenoid accumulation in the cuticle requires considerable carbon and energy input from the plants, they must contribute to the health or fitness of tea plants to be retained during the evolutionary history of tea.

## The Roles of Tea Leaf Cuticle in Biotic Stresses

Cuticular wax synthesis gene was induced by pathogen attack (Bourdenx et al., 2011; Zhao et al., 2020), raising the possibility that cuticle is involved in insects and pathogen defense. Recently, caffeine was detected from the tea leaf cuticle (Zhang et al., 2020, 2021; Chen et al., 2021), raising an interesting question that cuticular caffeine may be involved in insects or pathogens resistance. Due to its dual hydrophilic and lipophilic character, caffeine can freely penetrate cell-, tissue-, and organ-related barriers (Gundlach et al., 1992). In *Coffea* caffeine accumulation is boosted by insect- and pathogen-infestation (Filho and Mazzafera, 2000). In tomato, cabbage, and orchid exogenous application of caffeine can enhance their resistance to insects and pathogens (Hollingsworth et al., 2002; Ashihara et al., 2008). Transgenic tobacco plants producing caffeine showed enhanced pathogen resistance (Kim and Sano, 2008). The caffeine content in *Coffea arabica* showed a positive correlation with their resistance to *Colletotrichum coffeanum*-induced coffee berry disease (Biratu et al., 1996). These data demonstrated that caffeine indeed participates in herbivore or pathogen resistance. However, the underlying molecular mechanisms remain elusive. Wang et al. (2016) reported that tea caffeine level was increased by the infection of *Colletotrichum fructicola*, caffeine strongly inhibited mycelial growth *in vitro* by affecting mycelial cell wall integrity and PM permeability. Li et al. (2016) found that foliar application of exogenous caffeine in tea tree can enhance lipoxygenase activity and endogenous jasmonic acid content, and decrease *C. gloeosporioides*-induced necrotic lesions. These findings provide novel insights into the molecular mechanisms of caffeine in plant defense response. The tea cuticular caffeine could serve as the first line of defense in efficiently repelling pests and pathogens (Uefuji et al., 2005; Kim and Sano, 2008).

Besides caffeine, triterpenoids are mostly considered to have roles in plant protection and defense from pathogens or herbivores due to their inherent antimicrobial, antifungal, antiparasitic, insecticidal, and anti-feedant properties (Augustin et al., 2011; Osbourn et al., 2011). Zhu et al. (2018) found that the cuticle from the mature tea leaves is dominated by triterpenoids and sterols which are absent from that of the tender tea leaves. Zhou et al. (2019) also reported that triterpenoid ester content increased with leaf maturation. It is worth noting that the triterpenoid distribution pattern is in sharp contrast with caffeine, which is higher from the tender leaves than that of mature leaves (Ashihara et al., 2008). The differential distribution patterns might suggest that caffeine plays larger defense roles in the tender tea leaves, whereas triterpenoids are the major defense compounds in mature tea leaves. The cuticular triterpenoids, like its counterpart of cuticular caffeine, could also serve as molecular signatures during the initial phase of the interaction between pathogens, herbivores, and their plant host. Under this scenario, the chemical defense imposed by the tea host may force insects or pathogens to apply avoidance or adaption strategies. In tea green leafhopper [*Empoasca (Matsumurasca) onukii Matsuda*], a polyphagous phloem feeding specialist pest of tea plants in Asian tea growing regions (Qin et al., 2015), the nymphs and adults suck phloem sap of tender leaves rather than mature leaves and the female adults lay eggs within the stem of tender shoots. *Ectropis oblique hypulina Wehrli*, a chewing tea insect, only feeds on the tender tea leaves. These observations suggest that caffeine may play minor roles against green leafhopper and *E. oblique* infestation. To support this note, it has been reported that tea caffeine levels are not affected by green leafhopper (Zhao et al., 2020). It requires further clarification if these feeding preferences are related to triterpenoid avoidance. In contrast, anthracnose is only found on mature leaves but not tender leaves of tea plants (Wang et al., 2016). Since a reduced endogenous caffeine content is positively correlated with increased susceptibility of tea to *C. gloeosporioides*, and the mature leaves show lower caffeine contents compared with that of tender leaves (Ashihara et al., 2008), the data suggest that caffeine, rather than triterpenoids, play major roles in the defense of this fungal pathogen. Overall, these data suggest that different insects and pathogens could develop different tolerance to caffeine or triterpenoids during their coevolution history.

## The Contributions of Leaf Cuticle for Tea Quality

Tea leaf cuticle could affect tea quality in several ways: (1) Cuticle can affect the processing properties of tea leaves. Generally, the thicker the cuticle, the more brittle it is. This not only affects the processing properties of tea leaves but also the appearance of the processed tea which is an important quality factor; (2) postharvest withering treatment reduces leaf water content, thus, facilitating metabolite transformation. It is an essential step to shape the quality of made tea. As an efficient transpiration barrier, tea leaf cuticle affects the postharvest withering dynamic and thus could affect postharvest metabolome remodeling and tea quality. Interestingly, the cuticular transpiration rates show large variations among different tea germplasm (Figure 6; Chen et al., 2021). It remains poorly understood how the withering dynamic changes affect metabolite transformation and tea quality. (3) It has long been speculated that tea leaf cuticle contributes to the aroma quality of made tea. The wax chemical characterization provides two pieces of evidence to support this speculation (Zhu et al., 2018). Firstly, aromatic precursors are found in the tea cuticle in the form of esters, including benzyl alcohol, phenethyl alcohol, 1-butanol, isobutanol, and 2-ethyl-1-hexanol; secondly, benzyl esters and phenethyl esters are only found in the tender leaf which is used to make tea, and absent in the mature tea leaf. During postharvest tea processing, the tea leaves could be disintegrated, which results in the release of hydrolase, lipase, or esterase onto the leaf surface; the ester bond could be cleaved; and the cuticle aroma precursors be released, thus contributing to the aroma quality of made tea. The black tea shows a richer aroma than that of green tea. The black tea processing includes a rolling step which leads to complete leaf disintegration, and this would facilitate aroma precursor release from tea cuticle. (4) As a hydrophobic layer, the cuticle could slow down the release of tea-soluble contents into infusion during tea brewing. An unintended effect of tea processing might be to break the integrity of the cuticle to facilitate metabolite release. White tea, the less processed tea which only includes two steps, namely withering and drying, could better preserve the cuticle integrity in the processed tea. Interestingly, white tea is well-known to be more resistant to brewing than other types of tea. This correlation may provide indirect indication of the roles of the tea cuticle for tea brewing.

## Concluding Remarks and Future Perspective

In the past several years, significant progresses have been made to understand the tea leaf cuticle. The tea cuticular waxes are constituted by 14 chemical classes including acids, 1-alkanols, aldehydes, alkanes, 1-alkanol esters, glycol esters, benzyl esters, phenethyl esters, phthalate esters, glycols, tocopherols, triterpenoids, sterols, and caffeine. The cuticular waxes from the tender leaf are constituted by VLCFAs; in contrast, the cuticular waxes from the mature leaves are dominated by a high percentage of triterpenoids and steroids. In response to environmental stresses, the tender leaf and the mature leaf show some common and distinct patterns for cuticle modification including *in vivo* wax synthesis or transport in epidermal cells, dynamic phase separation between the EWs and the IWs, *in vitro* polymerization, and retro transportation into epidermal cell wall or cytoplasm for further transformation. The adaxial and the abaxial leaf surfaces showed different transpiration barrier organizations; the adaxial IWs were the major transpiration barrier, whereas the abaxial EWs constitute another major leaf transpiration barrier. Cuticular transpiration barrier modification does not require the overhaul of all wax components. Instead, targeted deposition of specific wax compounds into individual cuticular compartments is sufficient to alter cuticular transpiration barrier properties. Tea cuticle may contain molecular signatures for the initial interactions with some pathogens and herbivores, which could determine the susceptibility or resistance of tea to these biological stresses; in addition, the tea cuticle may contribute to the quality of tea made through several different mechanisms.

**Figure 8:**
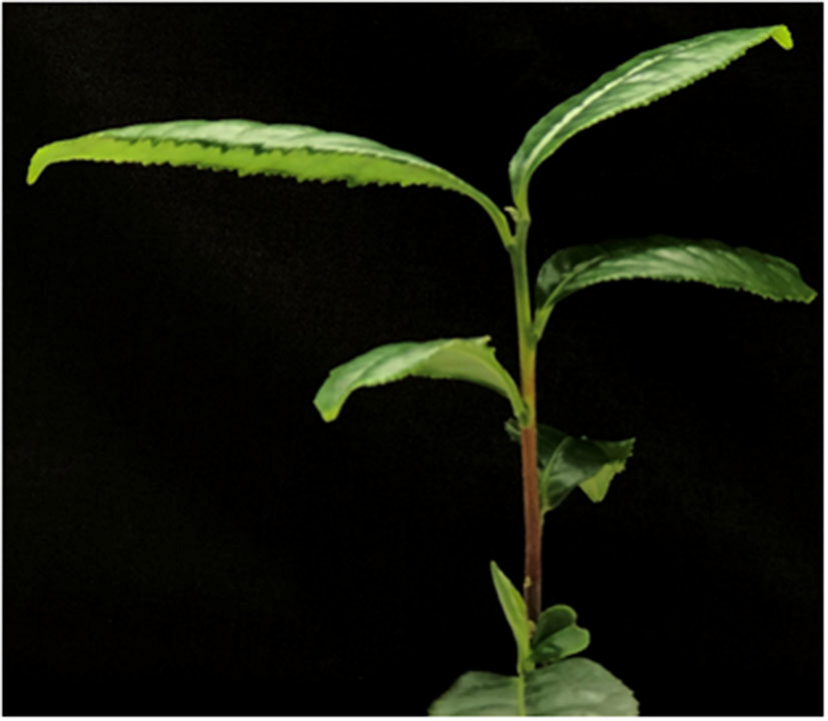
The stem from a growing twig of *Camellia sinensis*.

Even though these progresses were made in the past several years, there are many unresolved questions that should be addressed in the future. Below, the author would like to briefly discuss some of them:

1. From the mature tea leaf cuticle, ~43% of the wax mass detected by GC was not chemically identified (Zhu et al., 2018) due to their spectra being not in the National Bureau of Standards Mass Spectral Library. In the future, other analytical methods should be applied to resolve their chemical identity which is critical to understand structure–function relationships of the tea cuticle.
2. After the waxes are removed from the tea cuticle, the remaining polymer matrix still can maintain structural integrity. Generally, the polymer matrix of cuticle includes cutin, cutan, and polysaccharides (Nawrath, 2006; Domínguez, et al., 2011b). So far, the chemical composition of tea cuticle polyester has not been characterized, and in the future, this question should be addressed to have a holistic understanding of the mechanical characteristics of the cuticle.
3. What are the molecular mechanisms to regulate triterpenoid synthesis in epidermal cells during leaf development? In grape, Myb5b regulates triterpenoid synthesis, but the downstream targets are waiting to be identified. It still remains unclear whether a tea homolog of Myb5b would play similar roles as grape to activate triterpenoid synthesis.
4. How are triterpenoids transported across PM and cell walls? Are VLCFAs and triterpenoids transported through similar or distinct mechanisms?
5. Although the epidermis may contain detectable amount of chlorophyll (Suh et al., 2005), such low levels hardly make the epidermis trophic-independent. The cuticle synthesis in the epidermal cells represents huge carbon and energy investment for the plants. Thus, the epidermal cells are expected to rely on the underlying palisade cells or mesophyll cells for carbon and energy supply, either through the symplast pathway or apoplast pathway. One would expect that there should be active transport of photoassimilates between epidermal cells and the underlying palisade cells or mesophyll cells. Currently, it remains largely unknown what metabolites are transported into epidermal cells and how they are transported.
6. Besides tea leaves, the growing tender stems also are covered by a layer of cuticle. Initially, these stems showed a green color, then gradually became lignified and lost their green color. At this stage, the stem cuticle was replaced by a suberin layer (Figure 8). Currently, little information is available regarding wax composition, synthesis, and regulation from the tender tea stem. The developmental gradient of the tea stem cuticle could offer a useful system to study wax and suberin biosynthesis and regulation.

## Author Contributions

The author confirms being the sole contributor of this work and has approved it for publication.

## Funding

This work was supported by the Key-Area Research and Development Program of Guangdong Province (Grant No. 2020B020220004), the Guangdong Key Laboratory of Tea Plant Resources Innovation and Utilization/Tea Research Institute, the Guangdong Academy of Agricultural Sciences (Grant No. 2020KF06), the National Science Foundation of China (Grant No. 31870803), and the startup fund from Xinyang Normal University to MC.

## Conflict of Interest

The author declares that the research was conducted in the absence of any commercial or financial relationships that could be construed as a potential conflict of interest.

## Publisher's Note

All claims expressed in this article are solely those of the authors and do not necessarily represent those of their affiliated organizations, or those of the publisher, the editors and the reviewers. Any product that may be evaluated in this article, or claim that may be made by its manufacturer, is not guaranteed or endorsed by the publisher.
